# Propionate Affects Insulin Signaling and Progesterone Profiles in Dairy Heifers

**DOI:** 10.1038/s41598-018-35977-1

**Published:** 2018-12-04

**Authors:** A. Bedford, L. Beckett, K. Hardin, N. W. Dias, T. Davis, V. R. G. Mercadante, A. D. Ealy, R. R. White

**Affiliations:** Department of Animal and Poultry Science, Virginia Tech, Blacksburg, VA 24061 United States

## Abstract

Emerging data highlighting gut microbiome influences on health support evaluation of how microbial fermentation end-products influence postabsorptive systems. This study aimed to investigate the effect of increased propionate status on progesterone profiles and insulin sensitivity in dairy heifers. Eleven Holstein heifers, synchronized in estrus, were assigned to one of two continuous, 5-day IV treatments: sodium propionate (PRO; n = 5) or saline (CON; n = 6). These infusions culminated in a hyperglycemic clamp with daily blood samples for an additional 7 days. Plasma propionate concentrations increased over the first 9 h in PRO heifers, then decreased until day 3 when they matched CON heifers. Maximum plasma progesterone concentrations tended to be greater in PRO heifers than CON heifers (4.19 vs 3.73 ng/mL; *P* = 0.087). Plateau insulin concentrations in CON animals were significantly greater than those in PRO animals (249.4 ± 25.1 vs 123.9 ± 35.8; *P* = 0.008) with a trend for an increased insulin sensitivity index in PRO heifers compared to CON heifers (*P* = 0.06). These changes in plasma propionate clearance leading to increased progesterone response and changes in insulin sensitivity suggest a role for SCFA metabolism in reproductive hormone regulation.

## Introduction

Short chain fatty acids (SCFA) are the end products of fermentation of dietary carbohydrates by the anaerobic intestinal microbiota, and have multiple effects on mammalian energy metabolism^1^. Circulating SCFAs play a role in the regulation of both fatty acid and glucose metabolism^2–5^. These metabolic processes are vital to the energy status of an animal, and affect many downstream physiological processes. The connections between circulating SCFA supply and energy metabolism suggest more thorough investigation into how SCFA dynamics can be beneficially manipulated is warranted.

Although impacts of SCFA on energy metabolism are evident in several mammalian species^5^, they are particularly important in cattle. Over the past 60 years, improvements in the genetic capacity for milk production coupled with changes in nutritional management have been associated with a decline in fertility in lactating dairy cows^6^. While recent improvements have been observed, primarily due to reproductive technologies such as synchronization protocols, *in vitro* fertilization, and embryo transfer^7^, further room for improvement remains. High reproductive efficiency is a key component of sustainable dairy production and profoundly influences herd profitability^8^. Because fertility is linked with a number of physiological processes including energy metabolism, and insulin sensitivity, it is possible that shifts in nutrition that have occurred to support greater milk production have negatively contributed to reproductive status of dairy cows.

Acetate, propionate, and butyrate are the predominant SCFAs present in the rumen fluid, with their concentrations and relative proportions dependent on the amount and composition of feed ingested^9,10^. Altering SCFA dynamics has been minimally explored in relation to reproductive status but could be a viable strategy for improvements due to the known interactions among energy status, insulin sensitivity, and the hypothalamus-pituitary-gonadal axis^11,12^. Propionate is one of the main SCFAs produced in the rumen, generally found in the second greatest abundance. As the main precursor for gluconeogenesis, propionate is involved in energy homeostasis in ruminants. Although heifers were used in the current study, given the importance of glucose metabolism in milk production of the lactating dairy cow^13^, a deeper understanding of how propionate dynamics affect insulin response could lead to improved feeding approaches for these animals. Further, any changes in insulin response are likely to influence downstream hormone signaling, including progesterone production^14^.

Progesterone is responsible for regulating a number of physiological processes related to reproduction including ovulation, implantation, and pregnancy. In dairy cattle, increased plasma progesterone levels have been associated with improved conception rates^15,16^, maintenance of pregnancy^17^, and postpartum return to cyclicity^18^. Due to an elevated feed intake resulting in increased liver blood flow, lactating dairy cattle have increased steroid metabolism^19^. This could affect circulating progesterone levels, thus increasing the importance of understanding the influence of post-absorptive SCFAs.

The post-absorptive responses to SCFAs are comparable among mammals, therefore developing an understanding of increased propionate status in dairy cattle could lead to applications in other mammals. We hypothesize that due to the integral role of propionate as the major precursor for gluconeogenesis and as a regulator of energy homeostasis, increased propionate status will positively affect circulating progesterone concentration. The objective of this study was to investigate the effects of increased propionate status on insulin sensitivity and progesterone profiles in dairy heifers.

## Results

### Plasma Short Chain Fatty Acids

**Figure 1:**
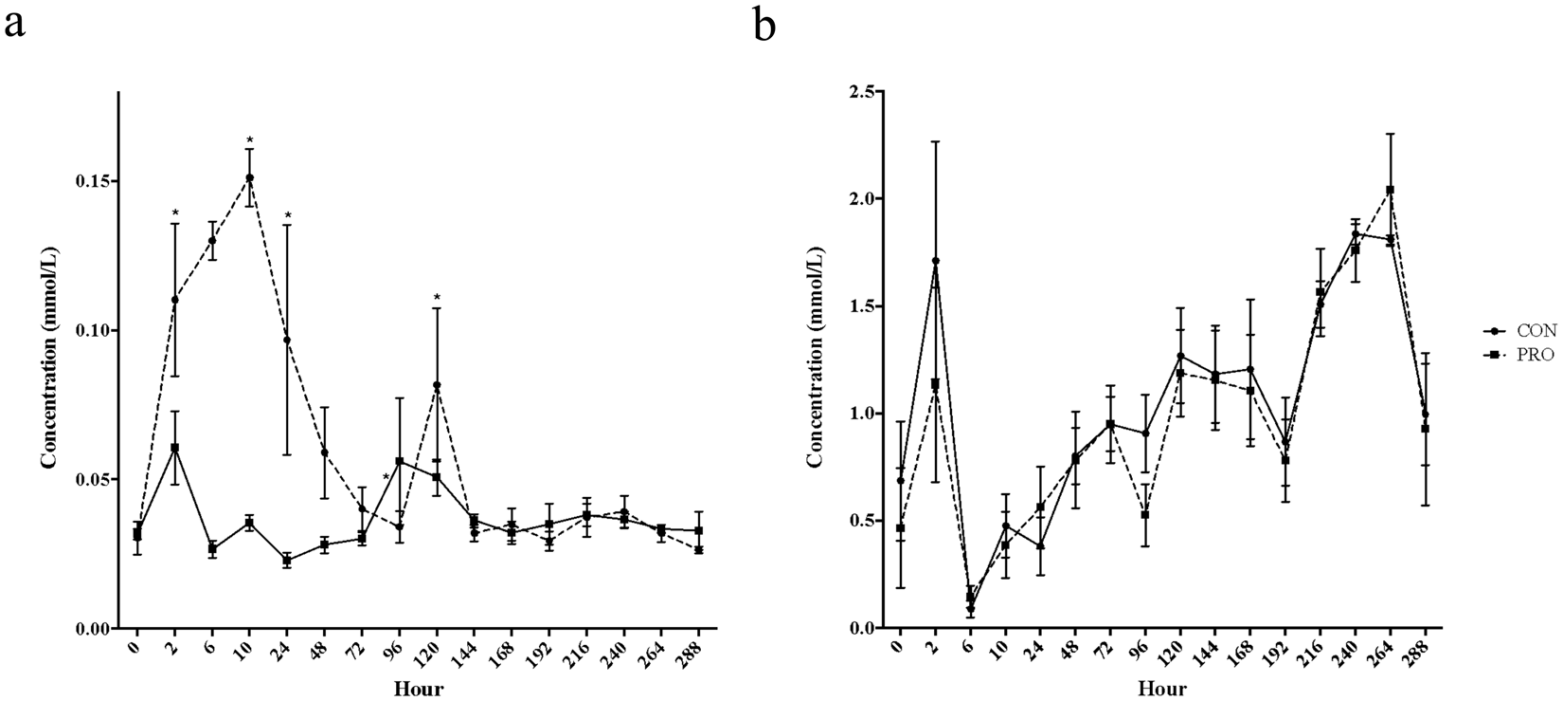
Plasma propionate and acetate concentrations (mmol/L) in PRO and CON heifers. Plasma profiles of propionate (**a**; mmol/L) and acetate (**b**; mmol/L) in PRO (dashed line) and CON(solid line) heifers over the entire experimental period. Timepoints where data differs significantly (*P* < 0.05) are denoted by*.

Plasma propionate and acetate levels are presented in Fig. 1. Concerning plasma propionate levels, there was a significant day by treatment interaction (*P* < 0.0001); propionate infusion led to significantly increased plasma propionate concentrations at 2, 10, 24, and 72 hours post initiation of infusion compared to CON animals (*P* < 0.01), with a peak at 10 h and declining concentrations thereafter. At 96 hours there was no difference between propionate concentrations of PRO and CON groups (*P* = 0.443). There was no significant effect of treatment on plasma acetate levels throughout the experiment (*P* > 0.10), but there was a significant day effect (*P* < 0.0001).

### Plasma Glucose

**Figure 2:**
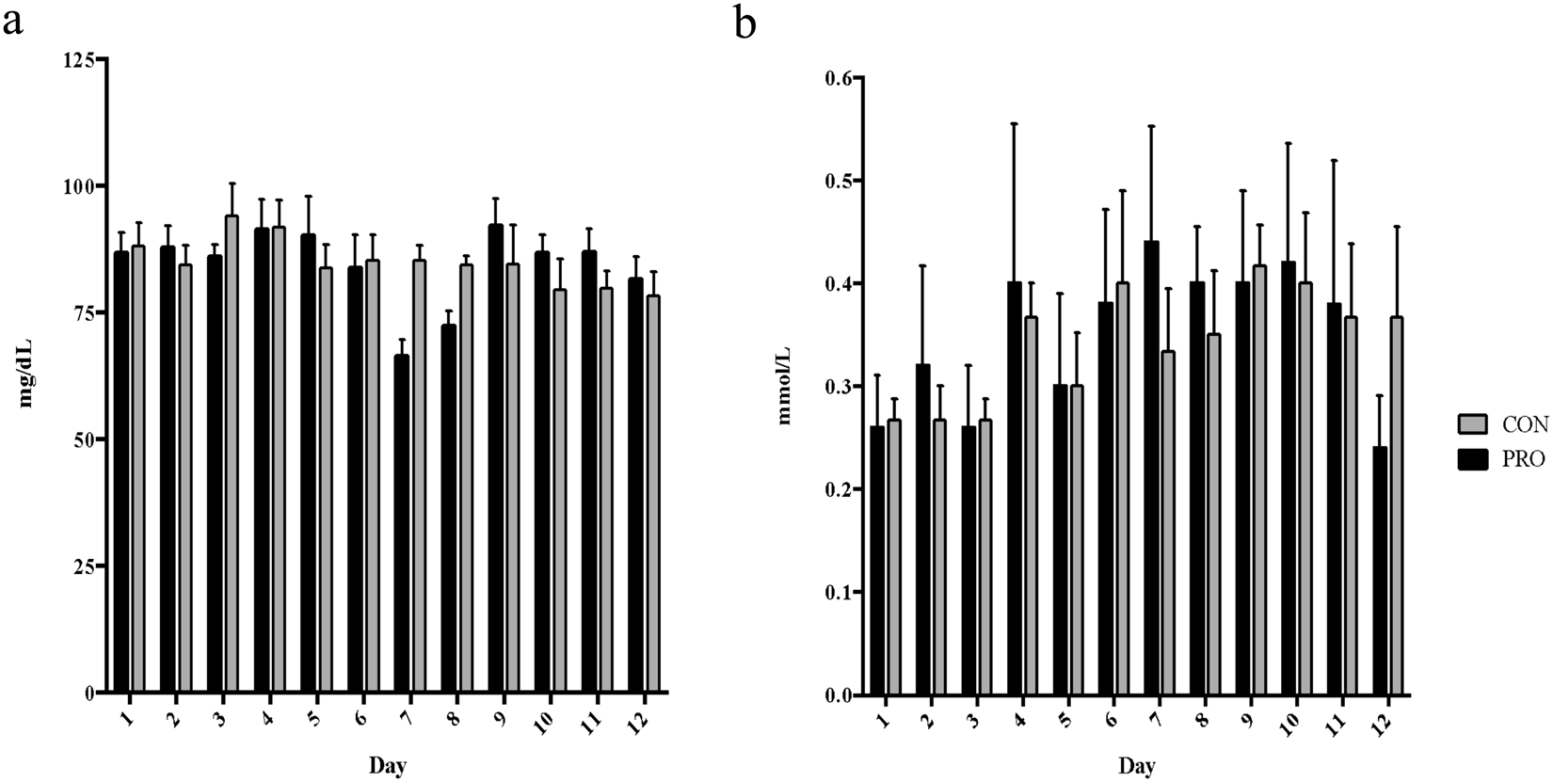
Blood glucose and beta-hydroxybutyrate concentrations in PRO and CON heifers. Whole blood daily measures of glucose (**a**; ng/dL) and beta-hydroxybutyrate (**b**; mmol/L) in PRO (■) and CON () heifers over the entire experimental period. Day 1 samples were those taken at 24 h after infusion start.

Plasma glucose concentrations are presented in Fig. 2a. There was no significant treatment effect on plasma glucose concentrations (*P* > 0.05); however, a significant day by treatment interaction (*P* = 0.041) suggested that glucose concentrations were significantly decreased on days 6 and 7 in PRO animals compared with CON animals.

### Plasma BHB

Plasma BHB concentrations are presented in Fig. 2b. Plasma BHB concentrations did not differ significantly with treatment, nor was there a significant day by treatment interaction (*P* > 0.05).

### Plasma Progesterone

**Figure 3:**
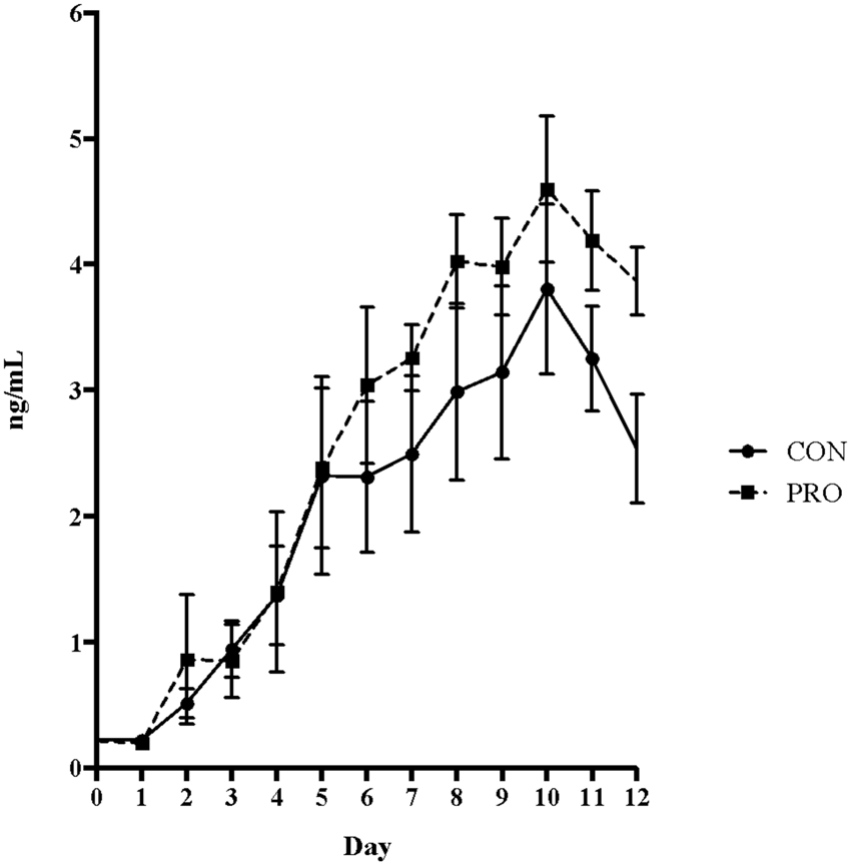
Plasma progesterone concentrations in PRO and CON heifers. Plasma profiles of progesterone (ng/mL) in PRO (dashed line) and CON (solid line) heifers over the entire experimental period.

Plasma progesterone concentrations are presented in Fig. 3. In both treatment groups, plasma progesterone concentrations rose in the days following completion of the OvSynch protocol. Although there were no significant differences in the time to rise or steepness of the progesterone profiles, there was a trend for significance in the magnitude, or maximum progesterone concentration, with PRO heifers having higher concentrations than CON heifers (4.19 vs 3.73 ng/mL; *P* = 0.087).

### Plasma Glucagon and Insulin

**Figure 4:**
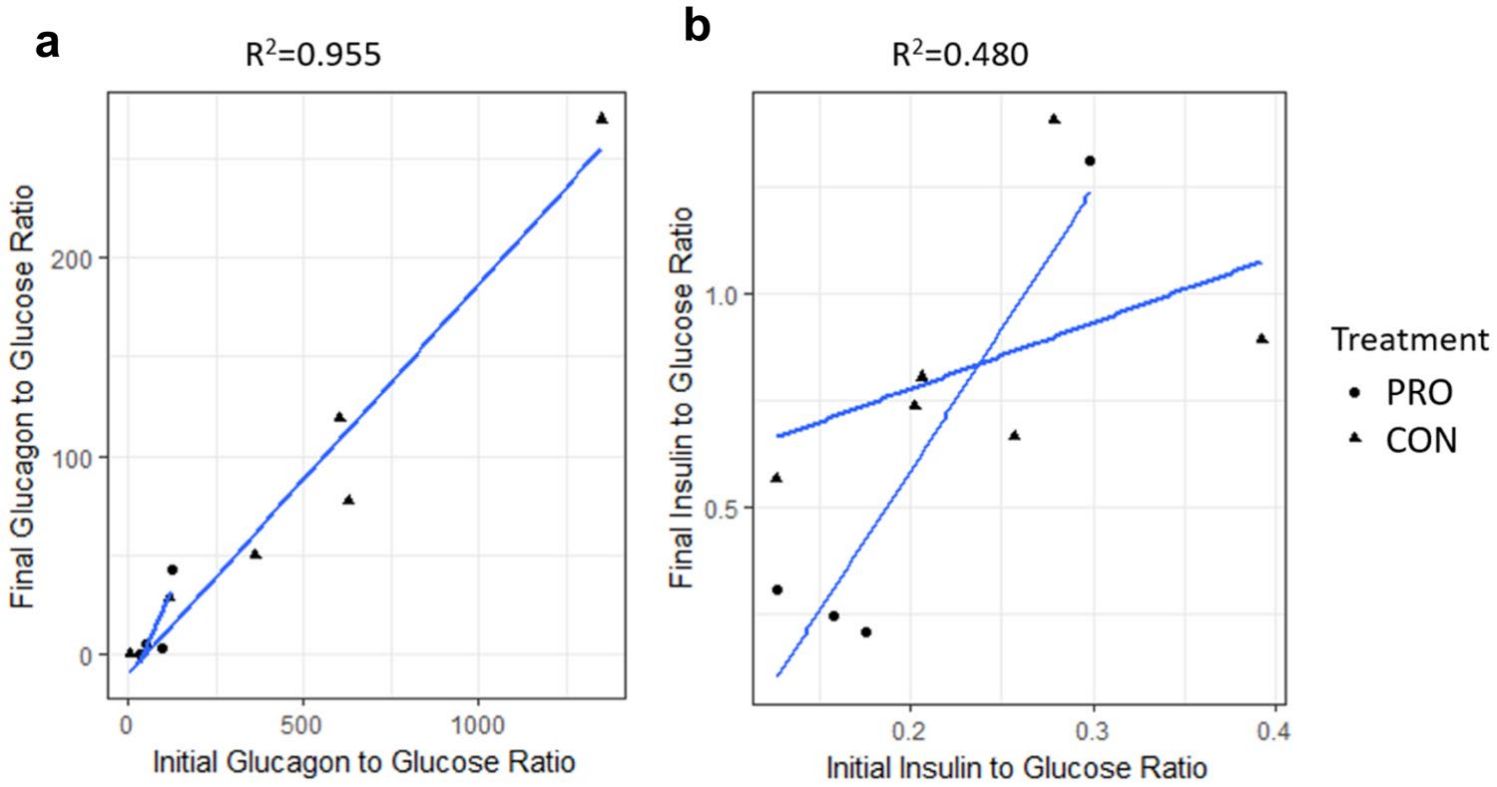
Ratio of final and initial glucagon or insulin concentrations to corresponding glucose concentrations. Circles represent animals on the CON treatment while squares represent animals on the PRO treatment. Least-square, linear regressions within treatment are shown to demonstrate within-treatment behavior. Pearson’s correlation coefficients for the overall relationship are included at the top of each plot.

Results from the hyperglycemic clamp are presented in Table 1. There were no differences in initial insulin, initial glucagon, plateau glucagon, initial glucose, plateau glucose, or glucagon response index between PRO and CON heifers (*P* > 0.10). Plateau insulin concentrations in CON animals were significantly greater than those in PRO animals (249.4 ± 25.1 vs 123.9 ± 35.8 μIU/mL; *P* = 0.008) and there was a trend for an increased insulin sensitivity index in PRO heifers compared to CON heifers (*P* = 0.06). The within-sample relationship between glucagon and insulin during the hyperglycemic clamp suggested a significant effect of change in insulin on change in glucagon (*P* = 0.004) with a moderate positive relationship (R^2^ = 0.56). The ratios of insulin and glucagon to glucose from the beginning and end of the clamp were assessed for correlations. Almost all of the variation in the glucagon to glucose ratio at the end of the clamp was explained by the variation at the beginning of the clamp (R^2^ = 0.96; Fig. 4a). For insulin, the starting ratio explained only half of the variation at the end of the clamp (R^2^ = 0.48; Fig. 4b).

**Table 1:** Response of PRO and CON heifers to a hyperglycemic clamp.

Item	PRO	CON
Initial^a^ Insulin (μIU/mL)	17.4 ± 2.4	18.8 ± 2.5
Initial Glucose (mg/dL)	85.0 ± 6.6	78.8 ± 4.0
Initial Glucagon (pg/mL)	6077.7 ± 11301.7	36556.6 ± 5650.8
Plateau^b^ Glucose (mg/dL)	178.5 ± 12.6	169.2 ± 12.1
Plateau Insulin (μIU/mL)	123.9 ± 35.8	249.4 ± 25.1
Plateau Glucagon (pg/mL)	3535.3 ± 8826.3	27189.1 ± 4413.2
Insulin Sensitivity Index	0.00086 ± 0.0001	0.00024 ± 0.0003
Glucagon Response Index	−7.0 × 10^−6^ ± 4.9 × 10^−5^	−8.1 × 10^−5^ ± 3.1 × 10^−5^

^a^Initial values are an average of three samples taken prior to glucose infusion.

^b^Plateau values are an average of six samples taken through the constant glucose infusion ending the clamp.

### Plasma Lipid Profiles

**Figure 5:**
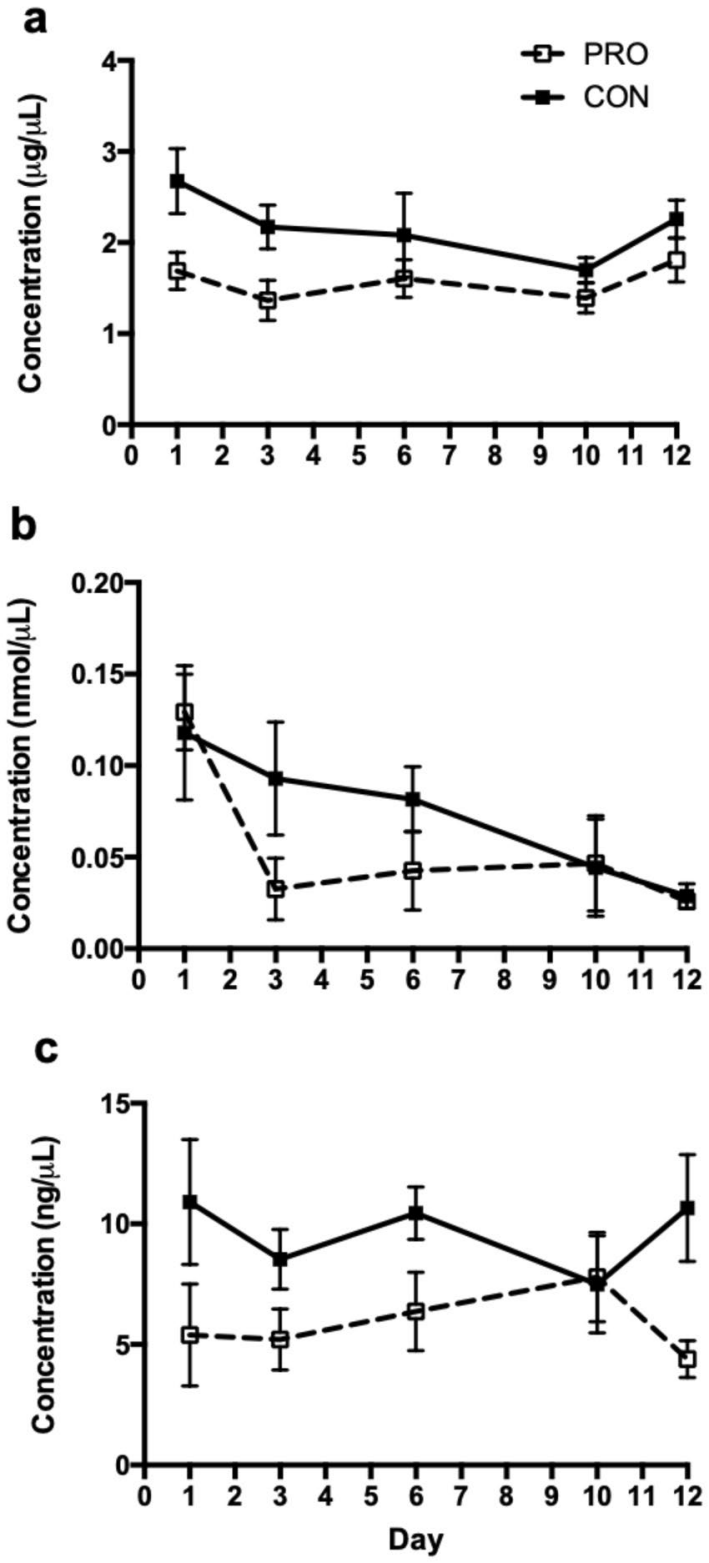
Plasma lipid metabolite concentrations in PRO and CON heifers. Plasma profiles of cholesterol (**a**; μg/μL), free fatty acids (**b**; nmol/μL), and triglycerides (**c**; ng/μL) in PRO (open box, dashed line) and CON (closed box, solid line) heifers through the experimental period.

Plasma lipid profiles are presented in Fig. 5. There was a trend for a treatment effect on plasma cholesterol concentrations (*P* = 0.07; Fig. 5a), with decreased concentrations in PRO animals. There was no significant day effect on plasma triglyceride concentrations (*P* > 0.10). There was no significant treatment effect on plasma FFA concentrations (*P* > 0.10; Fig. 5b), though there was a significant day effect (*P* = 0.004) with concentrations decreasing over the experimental period. There was a significant effect of day on plasma cholesterol concentrations (*P* = 0.02). There was a significant treatment effect on plasma triglyceride concentrations, with decreased concentrations in PRO animals (*P* = 0.03; Fig. 5c).

## Discussion

As the main substrates used for energy in cattle, SCFAs and their physiological dynamics are of great interest in the regulation of metabolism. With the variety of feedstuffs available, and the diversity of fermentation profiles of individual feedstuffs, theoretically, diets could be designed to manipulate SCFA profiles absorbed from the gut. This study used an intravenous propionate infusion to serve as an initial proof of principle for increased ruminal propionate absorption, and observed the effects of enhanced propionate status on insulin sensitivity and progesterone dynamics over an estrous cycle.

As expected, plasma propionate levels of PRO heifers increased over the first eleven hours of infusion while CON heifers remained at basal levels. Interestingly, plasma propionate levels in PRO heifers then decreased, reaching levels similar to CON heifers by day 3 of infusion. These results indicate that although propionate infusion initially raised circulating propionate concentrations, the metabolism of these heifers shifted to compensate for these increased levels, eventually returning plasma concentrations to normal. Peter and Elliot (1984) demonstrated rapid propionate clearance upon delivering an IV dose to Holstein cows^20^. Previous work evaluating hepatic SCFA clearance has demonstrated variation in and ability to adapt clearance capacity^21–23^. This metabolic shift to utilize extra available propionate gives merit to the hypothesis that increased propionate status affects downstream processes. No differences were observed in plasma acetate or BHB levels with the infusion of propionate, indicating that the utilization of these SCFA metabolites is regulated independent of one another. This is important when considering the unique metabolic utilization of each SCFA and how they impact the animal^24^.

Propionate is acknowledged to be the main precursor for gluconeogenesis in cattle^25^, and as such a change in plasma glucose was anticipated when propionate was infused. Although there was no overall treatment effect on plasma glucose, the drop seen in PRO heifers from day 6 to day 7 indicates a metabolic shift following the cessation of propionate infusion. Given that hyperglycemic clamps were performed on the day prior, this observed drop in glucose could be related to this procedure. Daily blood glucose levels in CON animals did not change from basal throughout the experiment. This indicates that the glucose administered during the hyperglycemic clamp to double basal circulating levels is likely not associated with the drop seen in PRO animals. Plateau insulin was significantly greater in CON animals compared to PRO animals, and this increase coupled with the implied decreased insulin sensitivity could have led to the reduced glucose observed in the following days. However, given the short half-life of insulin in circulation^26^, it is unlikely to be the cause of this glucose decrease. Further, the return of PRO heifer’s blood glucose plasma levels to basal on day 9 indicates a return to the metabolic norm. It is possible that this depression in plasma glucose following cessation of propionate infusion mirrors the shift seen at the beginning of infusion when propionate levels rose before returning to basal. We propose that the metabolism of cattle has a 2 to 3 day reaction lag in response to major alterations in post-absorptive energy status. This exact phenomenon has not been previously documented, as the majority of published literature focuses on the homeostasis state reached with experimental treatment and not the transition on to or off of treatment.

During the hyperglycemic clamp, PRO animals showed a tendency for increased insulin sensitivity. This was demonstrated by these animals maintaining reduced plasma insulin concentrations when glucose was infused to double the basal blood glucose concentration. Glucagon response index did not differ between groups, suggesting that the observed change is insulin response was not related to glucagon functionality. Previous work has established a role for SCFAs in glucagon and insulin dynamics, however, varying results have been reported. In mice, butyrate infusion was shown to improve insulin sensitivity^2^. In sheep, propionate infusion was shown to increase both circulating glucagon and insulin^27^. In cattle, ruminal propionate infusion showed a reactive increase in circulating insulin concentrations^28^; however, it was speculated that SCFAs do not have physiological implications for control of plasma insulin^29^. Although these early studies observed insulin and/or glucagon release into circulation, they did not perform any measures of insulin sensitivity.

In the present study, we did not observe any difference in basal plasma insulin or glucagon concentrations with propionate infusion. However, our initial quantifications did not occur until the final day of infusion. It is possible that plasma insulin and glucagon levels, much like propionate concentrations, had normalized at this time compared to the onset of propionate infusion. In multiple studies, chromium propionate has been demonstrated to increase insulin sensitivity in cattle^30,31^. Although chromium itself is known to potentiate the action of insulin in insulin sensitive tissues^32^, it is possible that propionate also contributed to the insulin and glucagon dynamics observed in these studies, as seen here. The increased insulin sensitivity observed here may also have had an effect on progesterone profiles due to the influence insulin has on the hypothalamus-pituitary-gonadal axis.

Although we observed increased insulin sensitivity in PRO animals, we saw no corresponding change in glucagon response. Meaning that while PRO animals required less insulin to manage their doubled circulating glucose levels, there was no corresponding shift in the responsiveness of glucagon. This result suggests that propionate does not modulate glucagon to the same degree as it does insulin. Previous work has shown single IV injections of propionate causing release of glucagon into circulation^33^, however, a prolonged propionate infusion was not observed. It is possible that heifers in the current study had an initial glucagon peak at the infusion start that returned to basal as the infusion continued resulting in no observable change in basal glucagon at the time of the clamp. Ruminal infusions of propionate have been shown to have no effect on plasma glucagon^34^, which supports the results observed here. We observed a moderate relationship between change in insulin and change in glucagon, with approximately half the variation in glucagon attributed to insulin, suggesting internal consistency in the response to supplemental glucose. The remainder of this variation may be related to treatment, which is supported by the relationship between the insulin to glucose ratios at the beginning and end of the hyperglycemic clamp, suggesting a change in the regulatory mechanisms of insulin by propionate.

Given the effect SCFAs can play in energy metabolism and thus, reproductive performance^5^, we measured circulating cholesterol, FFA, and triglycerides in our animals. We observed decreased circulating triglyceride and cholesterol levels in PRO animals. Plasma cholesterol concentrations have been shown to be positively correlated with plasma progesterone in the lactating dairy cow^35^, opposing what was observed here, suggesting an effect of propionate. In rodents and humans, SCFAs have been shown to reduce plasma concentrations of cholesterol^36,37^. Additionally, propionate has been shown to lower cholesterol synthesis by decreasing the enzyme activity of 3-hydroxy-methylglutaryl-CoA synthase and 3-hydroxy-3-methylglutaryl-CoA reductase^38,39^. Further, cholesterol acts as a substrate for the production of progesterone, therefore it is possible that the decrease observed in PRO animals is related to the increased progesterone profile. Either of these could explain the cholesterol decrease seen in PRO animals the current study. Reported effects of propionate on triglycerides are varied. In humans, orally supplemented propionate has been shown to increase triglyceride levels^40^, while a negative correlation between propionate and triglycerides has been reported in human males^41^. In general, SCFAs have been shown to decrease lipolysis in adipose tissue^42^, which could explain the reduced plasma triglycerides in PRO animals observed in this study.

The propionate profiles observed in this study, notably demonstrate how concentrations measured similar to basal, despite constant propionate infusion, indicates that using absolute concentration data may not be optimal for determining metabolic status. It is common in published literature to use circulating SCFA concentrations to draw metabolic conclusions for dairy cattle. However, this work demonstrates that using such pinpoint measurements may not accurately represent the complete picture. The concentration of circulating metabolites can change depending on rates of delivery to tissue, synthesis, clearance or exit rates, blood flow, and blood volume^23^. As a result, measuring SCFA concentrations in plasma likely has little to do with the actual supply to tissue. The unreliability of these measurements is likely heightened at times of high energy demand including growth, pregnancy, and lactation. For improved measurement of SCFA dynamics in circulation, estimating blood volume, influxes, and hepatic clearance may be necessary.

In the present study, propionate infusion led to greater plasma progesterone concentrations post-ovulation. To our knowledge, increased propionate status has not previously been linked with increased progesterone levels in dairy heifers. It is possible that the altered metabolic state, as seen by the return to basal propionate levels despite infusion, shifted towards increased gluconeogenesis. Circulating progesterone levels have been linked with nutritional status^43^, and this may be connected to the increased progesterone observed here. Increased circulating plasma progesterone could benefit reproductive efficiency by influencing follicular development and pregnancy rates^44^.

As previously mentioned, this increase in circulating progesterone may be related to the improved insulin sensitivity observed. Because the increased progesterone levels were observed on days beyond the cessation of propionate infusion, it is likely that this response is due to increased production and release from the corpus luteum. Improved response to insulin can positively affect modulation of luteal progesterone synthesis^45^. An additional contributor could be the reduced hepatic progesterone catabolism previously observed with improved insulin sensitivity^46^.

While the observations made here appear as a promising starting point for improving reproductive performance, delivery via jugular infusion is not practical in industry. One option could be the formulation of diets to increase propionate absorption from the rumen. In order for this to be possible, a deeper understanding of rumen SCFA fluxes is warranted. Measurements of rumen SCFA concentrations suffer from similar limitations as plasma concentrations: single point measurements do not account for fluxes or conversion rates. It could also be beneficial to give strategically timed propionate injections or boluses. This could easily be incorporated in an estrus synchronization protocol. However, the impact of one single injection, and the relationship between delivered and absorbed propionate would need to be determined first. Further, with the elucidation of rumen SCFA dynamics, the development of a probiotic supplement containing propionagenic bacteria may be of interest. Ultimately, for the most practical applications of this study, a deeper understanding of the mechanisms modulating post-absorptive propionate supply is required.

## Conclusions

Together, the results of the present study show a role for propionate in mediating insulin sensitivity and reproductive status. Future work with cattle should involve improving our understanding of SCFA dynamics and investigating practical applications that lead to increased propionate absorption. Further, work establishing the optimal production periods to apply these strategies for improved reproductive performance should be performed. These observed effects of propionate may also be beneficial for human health with potential applications in fertility, pregnancy, and treating diabetics.

## Materials and Methods

### Animals & Infusions

All procedures used in this study were approved by and performed in accordance with the guidelines and regulations enforced by the Virginia Tech Animal Care and Use Committee (IACUC #17-083). Twelve Holstein heifers aged 9.72 ± 0.9 months weighing 303 ± 36.6 kg, were paired by body weight and assigned to one of two treatment groups: propionate (PRO) or control (CON). Prior to the start of the treatment period, all animals were subject to an OvSynch + CIDR protocol. Briefly, animals were given a GnRH injection (0.1 mg, IM; Cystorelin, Factrel, Zoetis Inc., NJ, USA) and had an Eazi-Breed CIDR (1.38 mg of progesterone; Zoetis Inc., NJ, USA) inserted 11 days prior to the beginning of the experiment. The CIDRs were removed 3 days prior to the beginning of the experiment, in conjunction with a PGF2a injection (25 mg, IM; Dinoprost, Lutalyse, Pfizer Animal Health, NJ, USA). A second GnRH (0.1 mg, IM) injection was given at the time of catheterization (day 0 of the treatment period). In all animals, two indwelling jugular catheters (2 mm ID, 1 mm OD, micro-renathane, Braintree Scientific, Inc., Braintree, MA) were placed, one for infusion (60 cm in length), one for sampling (45 cm). Catheters were placed so that the tip of the sampling catheter was approximately 20 cm caudal to the tip of the infusion catheter to ensure sampled blood transited through the circulatory system prior to sampling. The PRO animals received an infusion of sodium propionate (P1880, Sigma-Aldrich, MO, USA) at a rate of 61.6 mmol/hr, while CON animals received sterile saline at a rate to match the volume of propionate infused. Infusions were delivered using Plum A + Infusion Systems (Abbott Laboratories, IL, USA). Saline and propionate solutions were sterilized and brought to a pH of 7.36 with NaOH prior to infusion. The treatment period lasted 12 days. Animals received their respective infusion for the first 5 days, a hyperglycemic clamp was administered on day 5, and the remaining 7 days were used for post-infusion monitoring. During the infusions, animals were housed in individual metabolic stalls, after which they were all moved to a common pen. In both housing settings, animals had *ad lib* access to feed and water. All animals were offered a standard heifer ration (Table 2) throughout the experimental period. The datasets generated and protocols used during the current study are available from the corresponding author on reasonable request.

**Table 2:** Diet Composition.

Item	Ingredient	% as fed
**Whole Ration**		
	Corn Silage (37% DM)	69.47
	Western Alfalfa (90% DM)	15.23
	Heifer Pellet (90% DM)	15.30
**Heifer Pellet**		
	Wheat Middlings	29.6
	Corn Meal	14.4
	Distillers Dried Grains	5.7
	Gluten Feed	19.7
	Soybean Meal	18.5
	Soybean Hulls	9.1
	Limestone	1.4
	Salt	1.1
	Trace Mineral Premix	0.06
	Vitamin E	0.1
	Vitamin A, D3, E Premix	0.08
	Rumensin	0.06
	0.06% Selenium Premix	0.2

### Blood Glucose and Beta-Hydroxybutyrate

Blood glucose and beta-hydroxybutyrate (BHB) were monitored daily using a Precision Xtra Blood Glucose and Ketone Monitoring System (Abbott Laboratories, IL, USA). An 8 mL blood sample was either drawn from the sampling catheter (days 0 to 5) or collected from the coccygeal vein (days 6 to 12) and blood glucose and BHB were immediately measured.

### Hyperglycemic Clamp

On the final day of infusion (experimental day 5), all animals were subjected to a hyperglycemic clamp. Twenty-four hours prior to the clamp procedure, animals were switched to feeding every 2 h to obtain a metabolic steady state. To perform this clamp, glucose (4.58 M, pH 7.36, sterile solution) was infused as a secondary line concurrent with propionate or saline through the jugular catheter. Basal glucose concentrations were determined immediately prior to glucose infusion in the same manner as described above. Glucose infusion rate was initially set to 2 mg/kg BW/min and was adjusted as necessary to achieve a circulating glucose concentration that were double the basal concentration. Blood samples (10 ml) were collected from the sampling catheter every 5 min and glucose concentrations were determined immediately to allow for glucose infusion rate adjustments. The remainder of these samples were transferred into heparinized blood collection tubes, centrifuged for 10 min at 10,000 rpm, and the plasma portion was transferred into a new storage tube and stored at −20 °C until further analysis. The hyperglycemic clamp was terminated once blood glucose concentration was maintained at double basal concentration for 30 minutes with minimal change in glucose infusion rate.

### Glucagon, Insulin and Progesterone Analysis

Plasma glucagon concentrations were measured in duplicate using a commercially available bovine competitive ELISA kit (Biomatik, Cambridge, ON, Canada). Plasma insulin and progesterone concentrations were measured in duplicate using a commercially available chemiluminescence assay (Immulite 2000 XPi Immunoassay System, Siemens Healthcare, CA, USA).

### Plasma Cholesterol, Free Fatty Acid, and Triglyceride analysis

Plasma cholesterol, free fatty acids (FFAs) and triglyceride concentrations were measured in duplicate using commercially available quantification kits according to manufacturer’s instructions (Cat. No. MAK043, MAK044, and MAK266, respectively; Sigma-Aldrich, St. Louis, MO, USA).

### Plasma Short Chain Fatty Acid Analysis

Plasma SCFAs were derivatized using the procedure previously reported by Kristensen^47^. Briefly, plasma samples were mixed with acetonitrile and 2-chloroethanol, vortexed, and centrifuged at 1,500 × g for 30 min at 4 °C. To resulting supernatant, NaOH and heptane were added followed by vortexing and phase separation. The lower phase was moved to a new vial, HCl, pyridine, and chloroethyl chloroformate were added, vortexing with each addition. Chloroform was then added to generate SCFA 2-chloroethyl esters following the addition of water. Following derivatization, samples were analyzed using a Thermo Electron Polaris Q mass spec (MS) in tandem with a Thermo Electron Focus Gas Chromatograph (GC) using XCalibur software (version 1.4). A Varian FactorFour capillary column VF-170ms (30 m, 0.25 mm, 0.25 um) was used. A total of 1 |L of sample was loaded with inlet temperature set to 225 °C on a split ratio of 80, running a constant flow of Helium carrier gas set to 1.2 mL/min. The GC was initiated at 75 °C, ramped at 5 °C/min to 135 °C, then at 40 °C/min to 225 °C. The MS was programmed to run in positive SIM mode collecting in three consecutive segments m/z pairs for acetate (43, 47), propionate (57, 59), and butyrate (71, 73) in that elution order. The processing method used to integrate the area under the curves for each m/z utilized the ICIS algorithm.

### Statistical Analysis

All statistical analysis was performed using R v. 3.4.0 (R Core Team, 2016).

Plasma SCFAs, glucose, cholesterol, free fatty acids, triglycerides, and BHB levels were analyzed using a mixed-effect model:

$${Y}_{ijk}=u+{a}_{i}+{d}_{j(i)}+{y}_{k}+a{y}_{ik}+(b+{o}_{j}){x}_{ij}+{e}_{ijk}$$

where *Y*_*ijk*_ is the metabolite concentration at time *k* on the *j*th animal assigned to treatment *i*, *u* is the overall mean, *a*_*i*_ is the fixed effect of diet *i*, *d*_*j(i)*_ is the random effect of heifer *j* within diet *i*, *y*_*k*_ is the fixed time effect, *ay*_*ik*_ is the fixed interaction effect between time and diet, and (*b* + *o*_*j*_)*x*_*ij*_ is the common regression coefficient (*b*) for initial metabolite concentration of animal *j* on treatment *i* (*x*_*ij*_), adjusted for slope deviation associated with treatments (*o*_*j*_), and *e*_*ijk*_ is the random error associated with the jth heifer assigned to the ith diet at time *k*. For each metabolite, several covariance structures were tested (compound symmetry, first order autoregressive, first order ante dependence covariance, and unstructured) and the covariance structure resulting in the lowest AICc was used for analysis of each response variable. First order autoregressive covariance was ideal for the acetate and BHB models, and compound symmetry was used for the propionate, triglyceride, free fatty acid, and cholesterol analysis.

Plasma progesterone levels were analyzed by first fitting a logistic curve to progesterone concentration data for each animal of the form:

$$[P4]=a/(1+bx\,{e}^{-time/c})$$

Where *[P4]* is the predicted progesterone concentration at any given time point (*time*), *a* reflects the curve slope, *b* reflects the curve steepness, and *c* is the horizontal asymptote. Because animals were split into two groups to facilitate timely completion of the hyperglycemic clamps, two experimental groups were used. The hyperglycemic clamp data for each animal was analyzed using a mixed effect model with a fixed effect for treatment and random effect for group. The progesterone curve parameters were analyzed because we wanted to describe the shape of the animals’ progesterone behavior rise during the estrus cycle. It would have been possible to analyze this data as a repeated measures model but the results of such an analysis would focus exclusively on differences at discrete time points, which have less meaning in this context than the average shape of the behavior over time.

Data obtained from the hyperglycemic clamps included basal and plateau insulin, glucagon, and glucose concentrations. These concentrations were analyzed using a mixed effect model with a fixed effect for treatment and a random effect for group. As described previously, multiple covariance structures were tested for each response variable. Autoregressive covariance was identified as ideal for each hyperglycemic clamp variable. For further analysis of hyperglycemic clamp data, insulin sensitivity indices (ISI) were calculated as previously shown in^48^ by dividing the glucose infusion rate by the change in insulin concentration multiplied by the steady state glucose concentration. A similar value was calculated for glucagon, the glucagon response index (GRI), by dividing the glucose infusion rate by the change in glucagon concentration multiplied by the steady state glucose concentration. The change in insulin and change in glucagon were calculated by averaging the respective concentrations of the final three clamp samples then dividing by the initial value.
